# *Aerococcus urinae* infective endocarditis in a healthy young man: a case report

**DOI:** 10.1186/s44215-023-00073-y

**Published:** 2023-09-18

**Authors:** Akira Sugaya, Arata Muraoka, Masayuki Doi, Koji Kawahito

**Affiliations:** 1 Department of Cardiovascular Surgery, Gyotoku General Hospital, Hongyotoku 5525-2, Ichikawa, Chiba 272-0103 Japan; 2 Department of Cardiovascular Surgery, Jichi Medical University School of Medicine, Yakushiji 3311-1, Shimotsuke, Tochigi 329-0498 Japan

**Keywords:** Infective endocarditis, *Aerococcus urinae*, Aortic valve, Mitral valve

## Abstract

**Background:**

*Aerococcus urinae* infection is a rare clinical condition that causes urinary tract infections in older individuals. Infective endocarditis (IE) caused by *A. urinae* in healthy young men is extremely rare; however, the mortality rates of IE caused by *A.* *urinae* are high if appropriate surgical treatment is not performed.

**Case presentation:**

A 39-year-old healthy man was admitted to our hospital with complaints of fever, cough, and dyspnea. Blood cultures revealed gram-positive cocci, and echocardiography revealed a 20 × 12-mm vegetation on the aortic valve and perforation of the anterior mitral valve leaflet. He was diagnosed with IE and underwent aortic valve replacement and mitral valve repair after removal of the infective focus. Matrix-assisted laser desorption ionization time-of-flight mass spectrometry was used to identify *A. urinae*. The patient was discharged in good health after 6 weeks of ampicillin/sulbactam therapy.

**Conclusion:**

Prompt diagnosis and surgical removal of infectious foci with optimal antibacterial therapy are critical to protect patients from rare and potentially fatal IE caused by *A. urinae*.

## Background

*Aerococcus urinae* is a gram-positive coccus that commonly causes urinary tract infections in elderly individuals with organic disease [1, 2]. Similar to enterococci, aerococci possess antibiotic resistance features, and the mortality rate of infective endocarditis (IE) caused by *A.* *urinae* is high if appropriate surgical treatment is not administered. Although the identification of this bacterium has recently increased with the widespread use of matrix-assisted laser desorption ionization time-of-flight mass spectrometry (MALDI ToF–MS) [3], IE caused by *A. urinae* is extremely rare, especially in healthy young men [4, 5]. Herein, we report a case of IE caused by *A. urinae* in a young man without underlying diseases.

## Case presentation

A 39-year-old healthy man was admitted to our hospital with complaints of fever, cough, and dyspnea. He was diagnosed with pneumonia and treated with erythromycin and ampicillin/sulbactam (ABPC/SBT) for 2 weeks. Although his temperature decreased after antibacterial therapy, his cough and dyspnea persisted. Blood cultures revealed gram-positive cocci, and echocardiography indicated vegetation on the aortic and mitral valve perforations, leading to a diagnosis of IE. The patient was then transferred to our ward.

Physical examination revealed a systolic murmur at the apex of the heart and dyspnea on exertion. Blood tests revealed an elevated white blood cell count of 14,200/mL, C-reactive protein of 4.43 mg/dL, and brain natriuretic peptide of 147 pg/mL. Liver and renal function was normal, and there was no anemia or thrombocytopenia. Chest radiography showed enhanced bilateral hilar shadows, and blood cultures tested using MALDI-ToF–MS were positive for *A. urinae*; however, the urine culture was negative.

To identify the causative agent, MALDI-ToF MS was used according to the following procedure: Blood samples were analyzed using a blood culture autoanalyzer (BD Bactec FX; Becton Dickinson, New Jersey, USA). If a positive signal was detected, the sample was cultured on a TWIN Plate 9, BTB agar medium, or Brucella HK agar medium (Kyokuto Pharmaceutical Industrial Co. LTD, Tokyo). After inoculating the plate, a laser was applied to the sample to ionize the sample with a matrix reagent and measure the ToF–MS waveforms of the bacteria-specific rRNA-component protein composition. The waveforms were matched to those of standard strains registered in the library. The obtained score was described using the match rate, and a match rate of 20 or higher was considered a good identification accuracy. The analysis was performed using a Bruker Daltonics MALDI Biotyper® ver.5.0.0.0 (Bremen, Germany).

**Fig. 1:**
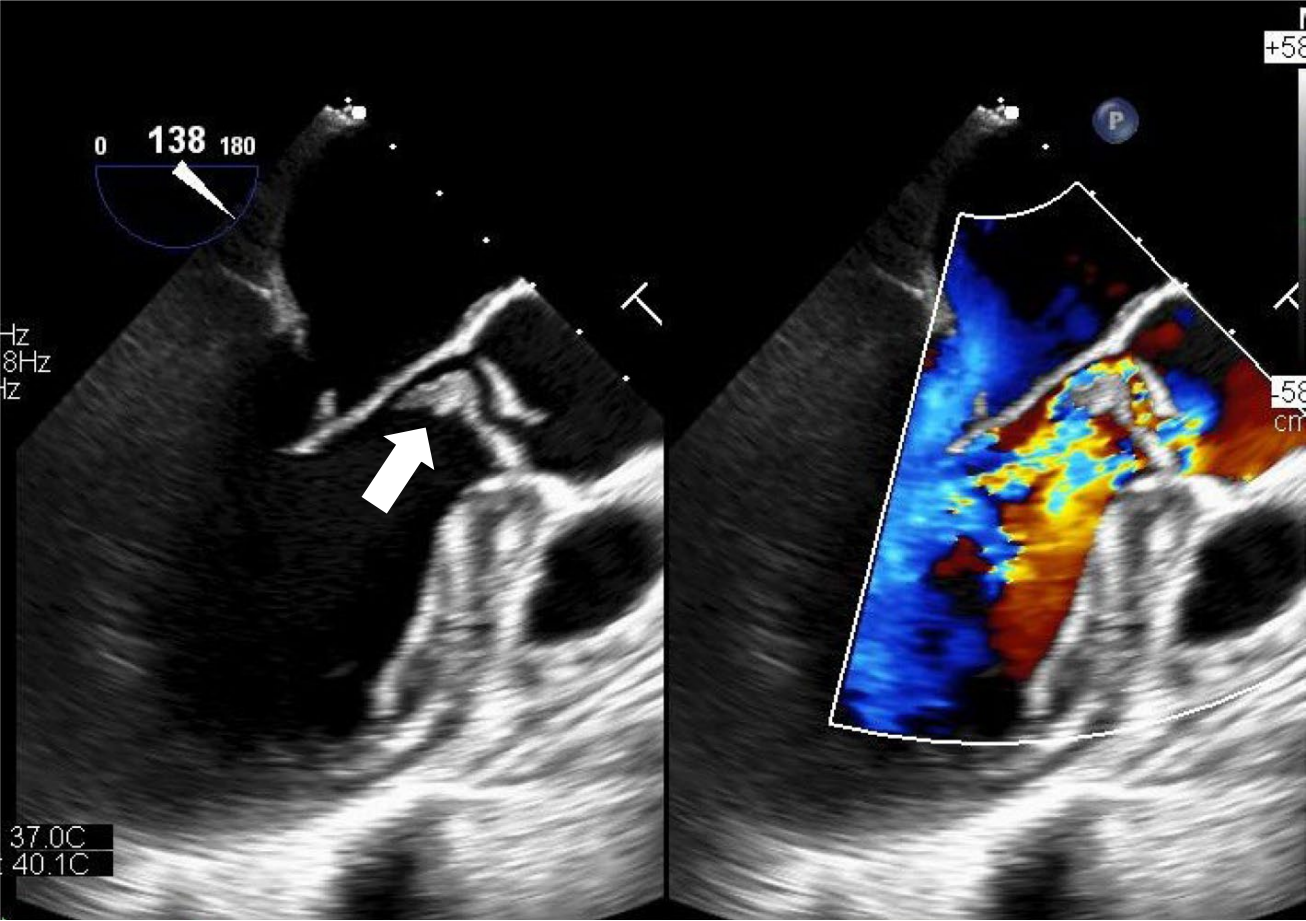
Transesophageal echocardiogram findings of the aortic valve. A large vegetation (20 × 12 mm) on the right coronary cusp leaflet, which reciprocates between the aorta and left ventricle with cardiac contraction, is observed (arrow) (left). Severe aortic regurgitation is noted (right)

**Fig. 2:**
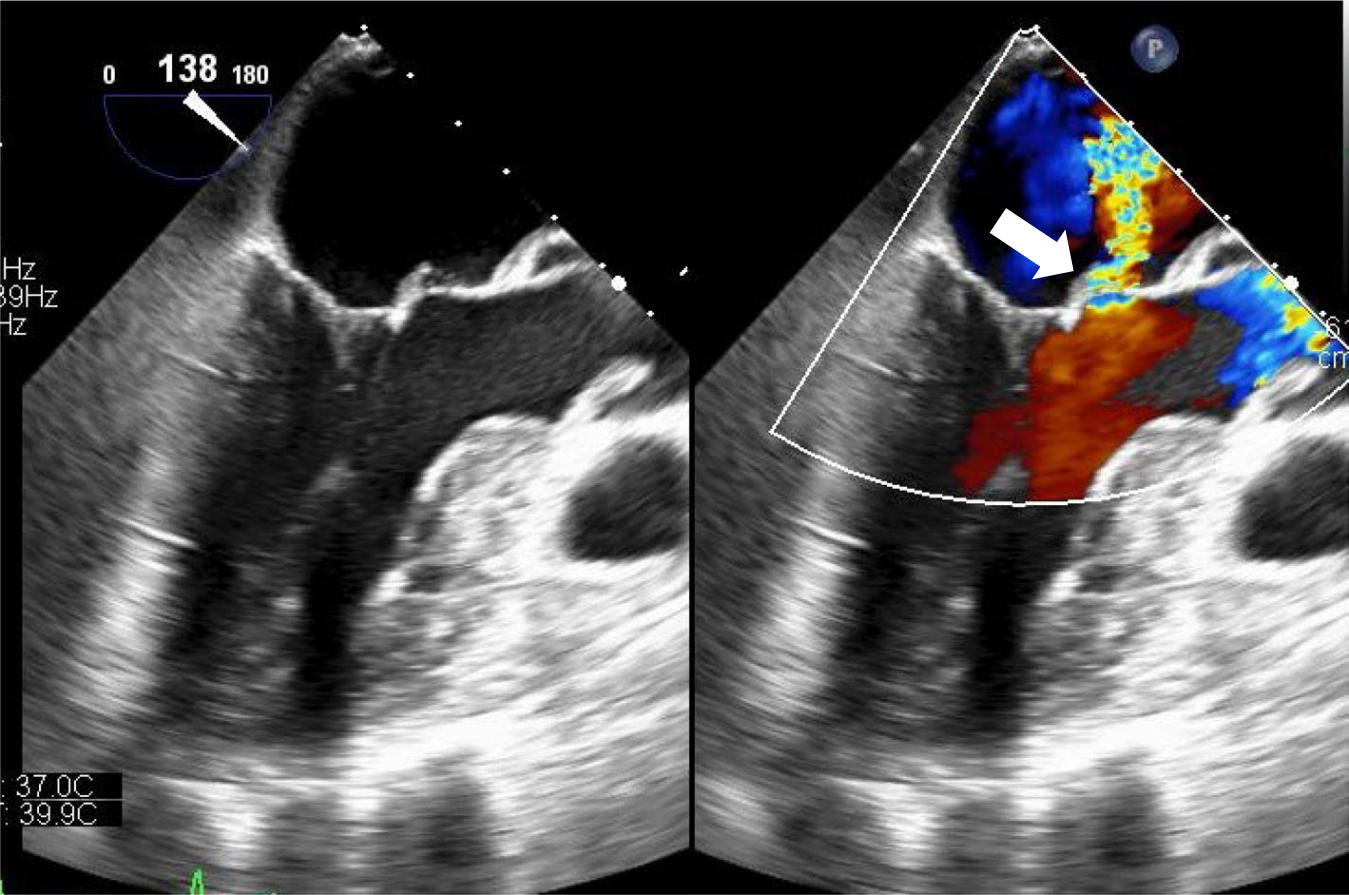
Transesophageal echocardiogram findings of the mitral valve. Perforation of the anterior mitral leaflet caused due to infection (right) (arrow)

Transthoracic and transesophageal echocardiograms revealed a large vegetation (20 × 12 mm) on the right coronary cusp leaflet, which reciprocated between the aorta and left ventricle with cardiac contraction, and severe aortic regurgitation was observed (Fig. 1). Echocardiography revealed perforation of the anterior mitral leaflet with vegetation and severe mitral regurgitation (Fig. 2). Whole-body enhanced computed tomography revealed no embolic findings.

**Fig. 3:**
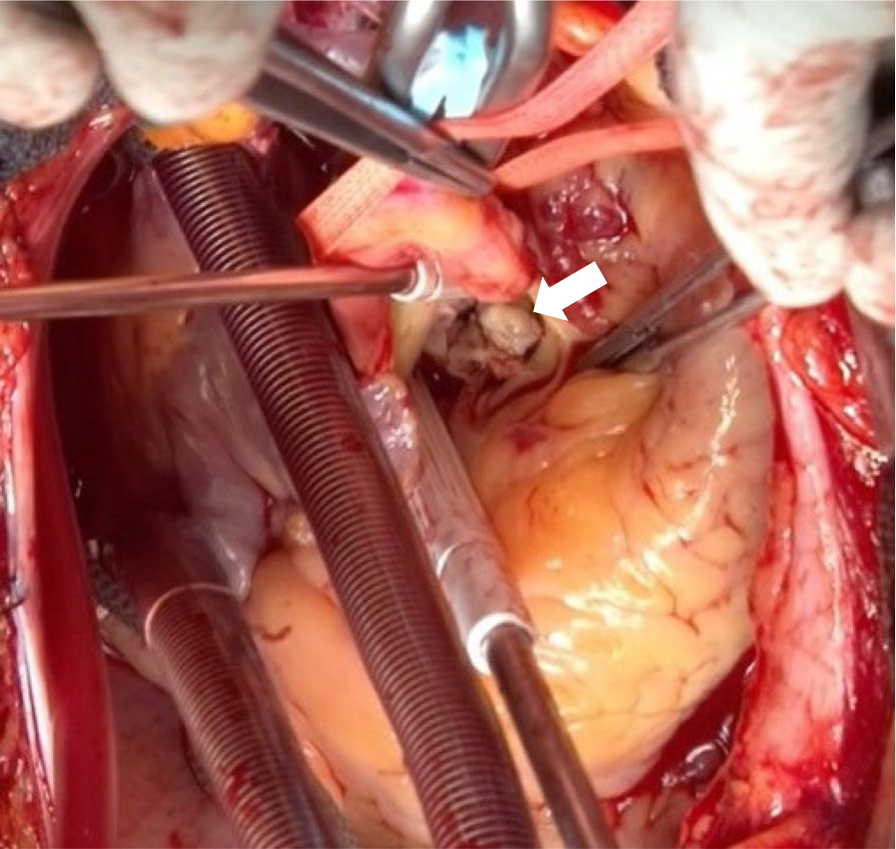
Intraoperative view of aortic valve. A large vegetation (20 × 12 mm) on the right coronary sac leaflet is observed (arrow). Additionally, the left and non-coronary cusp leaflets were destroyed due to the infection

**Fig. 4:**
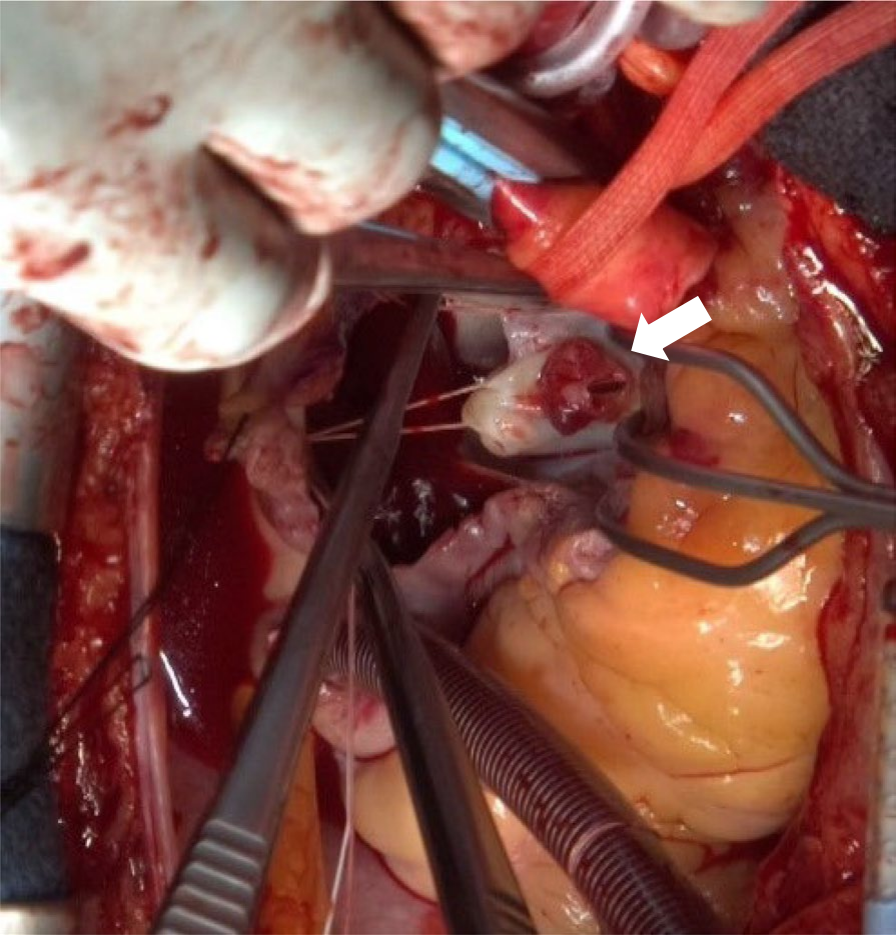
Intraoperative view of anterior mitral leaflet. Rash-like vegetation and perforation are observed in the anterior mitral valve leaflet (arrow)

The patient underwent urgent surgery using standard cardiopulmonary bypass through a median sternotomy. Intra operative findings revealed a large vegetation (20 × 12 mm) on the right coronary cusp leaflet, and all three valve leaflets were destroyed by the infection (Fig. 3). A vegetation perforation was observed in the anterior mitral valve leaflets (Fig. 4). The aortic valve was replaced with a 21-mm mechanical heart valve (SJM Regent™, Abbott Laboratories, Abbott Park, IL, USA), and the perforation of the anterior mitral leaflet was repaired with a bovine pericardial patch (Edwards Lifesciences Corporation, Irvine, CA) using 5–0 polypropylene continuous sutures after removing the vegetation.

The patient’s postoperative course was uneventful. The patient was discharged from the hospital after receiving ABPC/SBT for 6 weeks. Almost 1 year after the operation, the patient was healthy without any recurrence.

## Discussions and conclusions

*A. urinae* was first isolated as a rare cause of urinary tract infection, accounting for only 0.25% of urinary tract infections [1]. IE caused by *A. urinae* is extremely rare, with a prevalence of 3 per 1 million, and fewer than 50 cases have been reported in the literature [4]. However, *A. urinae* is increasingly being recognized as a human pathogen. Typically, patients with aerococcal IE are older men with underlying urinary tract abnormalities [6, 7]. In this case, a young man with no underlying disease presented with IE caused by *A. urinae* from an unknown invasive portal of entry. The patient had no sexual contact that could cause a sexually transmitted disease, and a post hoc examination did not reveal any organic abnormalities in the urinary tract.

*A.* *urinae* is a gram-positive alpha-hemolytic coccus, commonly misidentified as Streptococcus or Staphylococcus. Cluster colonies of *A. urinae* can simulate staphylococcal growth; however, aerococci do not share catalase positivity with Staphylococci. Although aerococci and enterococci share some features of antibiotic resistance, the correct determination of species was considered difficult in the past. Recently improved methods for species determination, especially the use of MALDI-ToF MS, have improved the identification and diagnosis of *A. urinae* [3–5].

MALDI-ToF–MS is divided into two processes: matrix-assisted laser desorption/ionization (MALDI), in which a laser is used to ionize the sample, and time-of-flight mass spectrometry (ToF–MS), in which ions are detected. Bacterial identification using MALDI ToF–MS is based on pattern matching between the protein mass spectrum of the tested bacteria and the mass spectrum data of the bacteria in the library. This method is considered an alternative to gene sequencing because of its rapidity and high concordance rate with 16S rRNA sequencing.

The mortality rate of IE caused by *A. urinae* can reach approximately 50%. Regarding the treatment and prognosis, the number of patients who survived with only medical treatment was low, whereas all patients who underwent surgery survived [6]. Although standard antibiotic therapy has not been established, antibacterial therapy using β-lactam antibacterial agents, such as penicillin, is commonly used. In some cases, aminoglycosides and vancomycin have also been used in combination [7, 8]. Certain studies have reported that gentamicin has a synergistic effect with penicillin and has often been used empirically with penicillin [8]. In this study, ABPC/SBT was selected based on the results of microbial sensitivity tests. Controlling the infectious source by surgical resection followed by antimicrobial therapy was effective.

Herein, we present a rare case of IE caused by *A. urinae* in a 39-year-old man with no underlying diseases. The patient underwent aortic valve replacement and mitral valvuloplasty and was discharged after 6 weeks of ABPC/SBT therapy. Prompt diagnosis and surgical removal of infectious foci, along with optimal antibacterial therapy, are crucial for the treatment of rare and potentially fatal IE caused by *A. urinae*.

## Data Availability

The datasets generated and/or analyzed in the current study are available from the corresponding author upon reasonable request.

## Abbreviations

IE: Infective endocarditis
MALDI-ToF-MS: Matrix-assisted laser desorption/ionization mass spectrometry
ABPC/SBT: Ampicillin/sulbactam
